# Association between salivary mature brain‐derived neurotrophic factor and psychological distress in healthcare workers

**DOI:** 10.1002/brb3.3278

**Published:** 2023-10-11

**Authors:** Atsuko Ikenouchi, Naomichi Okamoto, Shinsuke Hamada, Enkhmurun Chibaatar, Rintaro Fujii, Yuki Konishi, Ryohei Igata, Hirofumi Tesen, Reiji Yoshimura

**Affiliations:** ^1^ Department of Psychiatry University of Occupational and Environmental Health, Japan Kitakyushu Japan; ^2^ Medical Center for Dementia Hospital of University of Occupational and Environmental Health, Japan Kitakyushu Japan

**Keywords:** healthy healthcare worker, mature brain‐derived neurotrophic factor, psychological distress, saliva

## Abstract

**Introduction:**

Previous studies have suggested association between brain‐derived neurotrophic factor (BDNF) and the stress level of workers. However, no studies have investigated the potential of salivary mature BDNF (mBDNF) level as a noninvasive biomarker for psychological distress. This study aimed to explore the reliability of salivary mBDNF as a biomarker for psychological distress in healthcare workers. Furthermore, we examined the relationship between salivary and plasma mBDNF levels and their correlation with age, sex, body mass index (BMI), and exercise habits.

**Methods:**

Fifty‐one healthy healthcare workers (26 men) from the University of Occupational and Environmental Health, Japan, participated in this study. In this cross‐sectional study, participants provided demographic information. Psychological distress was assessed using the Kessler 6 (K6). Saliva and blood samples were collected, and mBDNF was measured by ELISA. Spearman's rank correlation coefficient was performed to analyze the relationship between mBDNF (saliva and plasma) and K6. Statistical analyses were conducted using Stata 17.0, and a significance level of *p* < .05 was applied.

**Results:**

The median K6 score was 1 (interquartile range [IQR]: 0–3). The median (IQR) salivary mBDNF was 1.36 (1.12–1.96) pg/mL, whereas the mean (standard deviation) plasma mBDNF was 1261.11 (242.98) pg/mL. No correlation was observed between salivary and plasma mBDNF concentrations or with the K6 score. Additionally, there were no associations between salivary or plasma mBDNF concentrations and age, sex, or exercise habits. Finally, an association between plasma mBDNF concentration and BMI was found only in univariate analysis.

**Conclusion:**

Our findings indicate that salivary mBDNF can be accurately measured noninvasively in healthcare workers. Within our study sample, salivary mBDNF did not demonstrate any correlation with K6 and plasma mBDNF. Future studies with a larger study sample and a diverse study population consisting of healthy participants and patients with psychiatric disorders are warranted.

## Graphical abstract

Salivary mature BDNF can be accurately measured non‐invasively in the saliva of healthcare workers. However, we found no correlation between salivary mature BDNF concentration and psychological distress or plasma mature BDNF concentration. This study focused on mentally healthy healthcare workers, limiting the generalizability of these results, and emphasizing the need for further investigation.

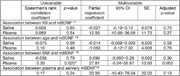

## 1 INTRODUCTION

Brain‐derived neurotrophic factor (BDNF) is a well‐studied neurotrophin, initially identified in 1982 (Adachi et al., 2014; Barde et al., 1982), with wide distribution in the brain (Ernfors et al., 1990) and various body tissues, including muscle (Murawska‐Ciałowicz et al., 2021), adipose tissue (Chaldakov et al., 2009), submandibular gland (Tsukinoki et al., 2006), lymphocytes (Wang et al., 2011), vascular endothelial cells (Meuchel et al., 2011), platelets (Yamamoto & Gurney, 1990), serum (Rosenfeld et al., 1995), and plasma (Okuno et al., 2011). Over the past three decades, it has garnered significant attention from clinicians and researchers due to its essential role in physiological brain functions, encompassing neurogenesis, synaptic plasticity, learning, and memory abilities (Egan et al., 2003; Hall et al., 2000; Kowiański et al., 2018; Lommatzsch et al., 2005; McAllister et al., 1999). BDNF levels can be influenced by factors, such as age, sex, body mass index (BMI), and exercise (Huang et al., 2014; Jasim, Ghafouri, Gerdle, et al., 2020; Lommatzsch et al., 2005; Okuno et al., 2011; Rojas Vega et al., 2006; Szuhany et al., 2015).

Serum and plasma measurements are well established, with serum BDNF levels (ng/mL) generally higher than plasma BDNF levels (pg/mL) due to the inclusion of BDNF stored in platelets (Fujimura et al., 2002; Mitoma et al., 2008; Okuno et al., 2011). Research on BDNF has primarily focused on serum rather than plasma (Lommatzsch et al., 2005). BDNF has the ability to cross the blood–brain barrier bidirectionally (Karege et al., 2002; Pan et al., 1998; Phillips et al., 1990). Plasma BDNF can potentially originate from neurons, glial cells, submandibular salivary glands, vascular endothelial (Nakahashi et al., 2000) and smooth muscle cells (Donovan et al., 1995), as well as activated monocytes, and lymphocytes (Kerschensteiner et al., 1999).

Mature BDNF (mBDNF) is synthesized from pro‐BDNF to mBDNF by several intracellular mechanisms (Foltran & Diaz, 2016). Pro‐BDNF induces long‐term depression via p75 neurotrophin receptors, promotes neurogenesis or development, and induces apoptosis or synaptic depression (Foltran & Diaz, 2016; Sakuragi et al., 2013). Conversely, mBDNF induces long‐term potentiation via tropomyosin receptor kinase B and promotes neurogenesis and development (Barker, 2009). Lower serum mBDNF levels have been observed in patients with depression compared to healthy controls, whereas serum pro‐BDNF levels do not differ significantly (Mikoteit et al., 2016; Yoshimura et al., 2014). Commercially available BDNF ELISA kits did not differentiate between mBDNF and pro‐BDNF, limiting studies specifically focused on mBDNF measurement (Lim et al., 2015; Lin et al., 2021).

BDNF is linked to pathological functions under stressful or harmful conditions. Thus, it plays an important role in neurodegenerative and psychiatric disorders (Angelucci et al., 2010; Nagahara & Tuszynski, 2011). Previous studies have observed a decrease in plasma and serum BDNF levels in patients with depression (Polyakova et al., 2015; Yoshimura et al., 2007), bipolar disorder (Cunha et al., 2006; Polyakova et al., 2015), and eating disorders (Nakazato et al., 2009; Yamada et al., 2012) and increases after treatment for these disorders (Nakazato et al., 2009; Yamada et al., 2012; Yoshimura et al., 2007). Plasma and serum BDNF have been identified as indicators of stress in both the general population and healthcare workers (Castillo‐Navarrete et al., 2023; Håkansson et al., 2017; He et al., 2018; Okuno et al., 2011). A recent study found that higher academic stress is associated with lower peripheral BDNF, with sensitivity to the duration of stress (Castillo‐Navarrete et al., 2023). Reduced serum BDNF levels have been observed in nurses experiencing insomnia (Furihata et al., 2020), and BDNF gene polymorphisms, such as Val66Met and rs6265, have been implicated in moderating occupational distress among Chinese healthcare workers (He et al., 2018; Jia et al., 2021). Individuals experiencing burnout due to occupational stress often experience low serum BDNF levels. Research suggests that elevated BDNF levels have a buffering effect on burnout under low stress conditions, but this protective effect diminishes as stress levels increase (He et al., 2020). BDNF is influenced by stress and exhibits sensitivity to stress type, duration, and timing (Bath et al., 2013).

Although peripheral BDNF levels play a critical role in understanding the biological mechanisms associated with psychological distress, research has been constrained by the reliance on blood samples. Conversely, saliva sampling offers a simple and noninvasive alternative to serum samples, as it can be easily collected under any circumstances without the need for specialized personnel. Although BDNF has been identified in saliva (Mandel et al., 2009, 2011), including its expression in the human submandibular gland (Saruta et al., 2012), the extent to which salivary mBDNF interacts with psychological distress in the healthy population, including healthcare workers, remains unclear. A recent study by Saruta et al. (2020) highlighted the significance of salivary BDNF during stress in healthy volunteers. Although these findings suggest a potential association between salivary mBDNF and psychological distress, and the possibility of salivary mBDNF serving as a biomarker, further investigation is still required.

Thus, this study aimed to investigate the potential use of salivary mBDNF as a reliable indicator of psychological distress in healthcare workers, which has not been previously documented. This study also examined the relationship between salivary and plasma mBDNF levels, along with their correlation with age, sex, BMI, and exercise habits.

## 2 MATERIALS AND METHODS

### 2.1 Participants

The study was conducted among healthcare workers at the University of Occupational and Environmental Health, Japan. Based on the results of preliminary experiments, power calculations were performed using EZR functions (Saitama Medical Center, Jichi Medical University) (Kanda, 2013). The power analysis, with a significance level of 0.05 and a power of 0.80, estimated the number of cases needed for the study to be 47. Volunteers were recruited using a bulletin board at the University of Occupational and Environmental Health, Japan. Informed consent was obtained from 51 participants. Healthcare workers between the ages of 18 and 65 were included in the study. Any participants with comorbid depression or other psychiatric or neurodegenerative diseases, hypertension, diabetes, dyslipidemia, malignancy, heart failure, renal failure, or other serious diseases requiring the treatment or presence of blood in saliva, were excluded from the study.

Participants were instructed to refrain from brushing their teeth and consuming any food/drink 1 h before sample collection. Participants were instructed to complete a questionnaire that included questions on age, height, weight, smoking status, alcohol consumption, exercise habits, presence of diseases requiring treatment, general health status, and Kessler 6 (K6) (Furukawa et al., 2008; Kessler et al., 2002, 2003; Sakurai et al., 2011). A structured interview using the Mini‐International Neuropsychiatric Interview was used to confirm the absence of psychiatric disorders (Sheehan et al., 1998).

One participant was excluded due to blood in their saliva. Fifty participants (25 men and 25 women) with no psychiatric disorders or deficient values were included. Among these participants, there were 32 physicians, 6 nurses, 4 pharmacists, 4 medical clerks, 3 psychologists, and 1 psychiatric social worker.

### 2.2 Assessment of psychological distress

The assessment of psychological distress was conducted using the Japanese version of the K6 questionnaire (Kessler et al., 2002), which has been validated in previous studies (Furukawa et al., 2008; Sakurai et al., 2011). The K6 is designed to screen for psychiatric disorders, including depression and anxiety, and serves as an indicator of the severity of mental issues, including psychological stress. It consists of six questions, with response options ranging from 0 (never) to 4 (always), reflecting the frequency of experiences during the past 30 days. The total score ranges from 0 to 24, with higher scores indicating greater psychological distress. A score of 5 or higher has been associated with the possibility of depression or anxiety disorder, whereas a score of 12/13 is used as the cut‐off point (Furukawa et al., 2008; Sakurai et al., 2011).

### 2.3 Procedures

Sample collection was conducted from 10:00 to 16:30. Prior to saliva collection, participants rinsed their mouths with distilled deionized water to remove oral debris, hydrated, and rested for 10 min. The participants were instructed to keep their eyes open and avoid conversation or any mental or physical stimuli that might stimulate salivation (Vrijen et al., 2017). To prevent degradation, all participants were instructed to drip unstimulated saliva (10 mL) into pre‐chilled protein low‐absorption type conical tubes. Venous blood was then collected in a plastic blood collection tube containing ethylenediaminetetraacetic acid disodium salt as an anticoagulant (Okuno et al., 2011). Saliva was centrifuged in a KUBOTA 5922 (KUBOTA) at 3500 rpm for 10 min at 4°C. The sample's supernatant (upper two thirds of the sample) was transferred to another tube and centrifuged in a KUBOTA 3520 (KUBOTA) at 15,000 rpm for 20 min at 4°C. Blood was centrifuged in a KUBOTA 5922 at 2000 rpm for 20 min at 4°C, and the supernatant (upper two thirds of the sample) was collected. These samples were frozen in a deep freezer at −80°C until assay.

### 2.4 Measurement of mature BDNF

Saliva and plasma mBDNF were measured using the Mature BDNF ELISA Kit Wako, High Sensitive (FUJIFILM Wako Pure Chemical Corporation) (Want et al., 2023) as follows. Briefly, standard solution and samples were dispensed into each well of the antibody‐coated plate after the removal of the plate protection solution and washing. After mixing, the plate was incubated at room temperature (20–25°C) for 2 h. After the reaction solution was discarded and washed, biotin‐conjugated antibody solution was added to each well and allowed to stand for 1 h at room temperature after mixing. After the reaction mixture was discarded and washed, peroxidase‐conjugated streptavidin solution was added to each well. The wells were incubated at room temperature for 30 min after mixing. After the reaction solution was discarded and washed, the luminescent reagent was dispensed into each well. After mixing, luminescence intensity was measured thrice in a 96‐well microplate reader. The range of the calibration curve was 0.205–50 pg/mL. The reactivity with recombinant human pro‐BDNF, NGF, NT‐3, and NT‐4 was <0.5%.

### 2.5 Coefficient of variation

Two types of coefficients of variation (*CV*) were used to assess the accuracy of ELISA results: intra‐assay *CV* and inter‐assay *CV*. The *CV* was calculated using the following formula: *CV* = standard deviation (*SD*)/*mean* × 100 (%). The intra‐assay *CV* evaluates the variation between individual data points within the same assay, involving the measurement of replicate samples on a single plate. The inter‐assay *CV* assesses the consistency between plates by measuring the variation between replicate samples measured on different plates. To ensure reliable results, the intra‐assay *CV* should be below 10%, and the inter‐assay *CV* less than 15%. In this study, 25 saliva and 25 plasma samples were each measured in triplicate using one 98‐well plate to calculate the intra‐assay *CV*. The inter‐assay *CV* was measured using three different plates. For the mBDNF assay using ELISA, the intra‐assay *CV* was 3.3% in saliva and 0.8% in plasma, whereas the inter‐assay *CV* was 10.2% in saliva and 4.6% in plasma.

### 2.6 Statistical analysis

The overall distribution of each variable was checked by plotting a histogram and kernel density estimation. BMI and plasma mBDNF exhibited a normal distribution, whereas age, K6, and salivary mBDNF demonstrated non‐normal distributions (Shapiro–Wilk test, *p* < .01). Descriptive data were presented as *means* (*SD*) for normally distributed variables and medians (interquartile range [IQR]) for non‐normally distributed variables.

Statistical significance of correlations between mBDNF (saliva and plasma) and K6, age, or BMI, as well as the correlation between salivary mBDNF and plasma mBDNF, was determined using Spearman's rank correlation coefficient. Each factor was analyzed using multiple regression analysis, and the adjusted *p*‐values for age, sex, BMI, and exercise habits were used as adjustment factors. The model for multiple regression analysis was assessed using histograms for the normality of residuals. The sex difference in mBDNF (saliva and plasma) was analyzed using the two‐sample Wilcoxon rank‐sum test. We calculated adjusted *p*‐values for adjustment factors using multiple regression analysis. We also calculated the *p*‐value and adjusted the *p‐*value for differences in mBDNF (saliva and plasma) with and without exercise habits. We also calculated the effect size (*r*) from the *z*‐value of the Wilcoxon rank‐sum test for each test of difference. For all analyses, tests were two‐tailed, and the significance level was set at *p* < .05. Statistical analyses were performed using Stata 17.0 (StataCorp), and the graph was created using Python 3.0.

### 2.7 Ethical considerations

This study was conducted in accordance with the tenets of the Declaration of Helsinki of 1975 (revised in 2008). The study was approved by the Ethics Committee of the University of Occupational and Environmental Health, Japan (R3‐082), and written informed consent was obtained from all participants.

## 3 RESULTS

Fifty participants (age: 22–61 years; median [IQR], 32 [29–37] years) were included in the analysis (baseline participants characteristics in Table 1). Participants exhibited a mean BMI of 21.8 (2.68) and a median K6 score of 1 (0–3) points. Twelve participants (24%) exercised at least twice a week. Salivary mBDNF levels were 1.36 (1.12–1.96) pg/mL, and plasma mBDNF was 1261.11 (242.98) pg/mL.

**TABLE 1:** Baseline characteristics of the participants.

	Total (*n* = 50)	Men (*n* = 25)	Women (*n* = 25)
Age	32 (29–37)	32 (31–35)	31 (27–42)
BMI	21.8 (2.68)	22.87 (2.80)	20.89 (2.20)
K6 score	1 (0–3)	1 (0–2)	2 (0–4)
Exercise habits (with/without)^a^	12/38	7/18	5/20

*Note*: Age and K6 were described by median (IQR), and others were *mean* (*SD*).

^a^Participants who exercised at least 2 days per week were defined as with exercise habits.

Abbreviations: BMI, body mass index; IQR, interquartile range; K6, Kessler 6; *SD*, standard deviation.

**FIGURE 1:**
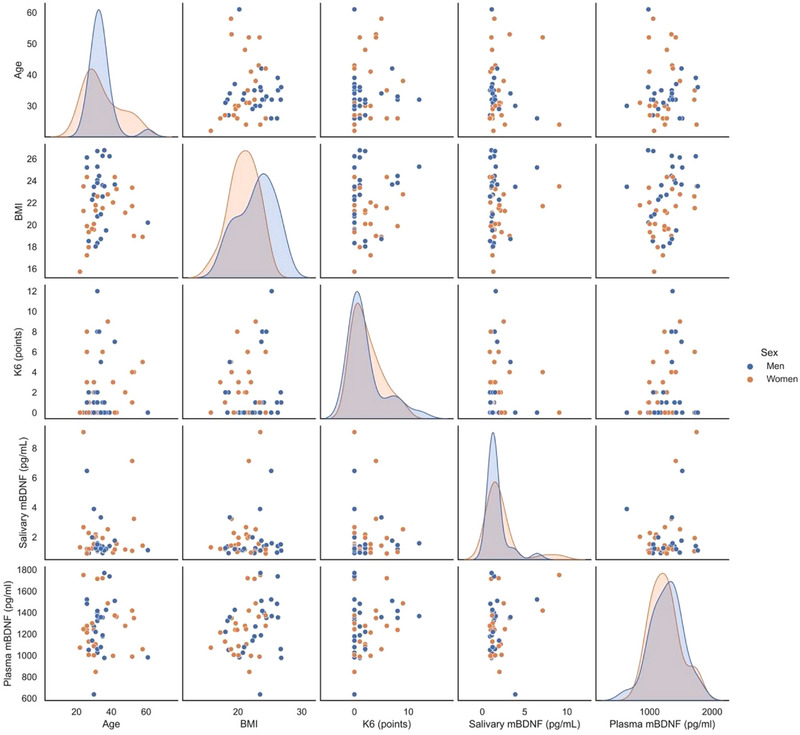
Scatter plot matrix (scatter plot and kernel density estimation) of brain‐derived neurotrophic factor (BDNF) association between each factor.

Spearman's rank correlation coefficient showed no correlation with K6 or age for either salivary mBDNF (K6; *p* = .85, age; *p* = .60) or plasma mBDNF (K6: *p* = .54, age; *p* = .48). No correlation was found between salivary mBDNF and BMI (*p* = .79); however, a correlation was found between plasma mBDNF and BMI in univariate analysis (*p* = .020). No correlation was found between plasma mBDNF and salivary mBDNF (*p* = .24). Multiple regression analysis showed similar results except for BMI. No correlation was found between plasma mBDNF and BMI in multivariable analysis (*p* = .052) (Table 2, Figure 1).

**TABLE 2:** Mature brain‐derived neurotrophic factor (mBDNF) association among each factor.

	Univariable		Multivariable			
	Spearman's rank correlation coefficient	*p‐*Value	Partial regression coefficient	95% CI	SE	Adjusted *p*‐value
Association between K6 and mBDNF^a^						
Saliva	0.028	.85	−0.027	−0.19 to 0.13	0.079	.73
Plasma	0.089	.54	12.95	−10.68 to 36.58	11.72	.27
Association between age and mBDNF^b^						
Saliva	−0.075	.60	−0.014	−0.068 to 0.039	0.026	.58
Plasma	0.10	.48	−1.053	−9.12 to 7.02	4.006	.79
Association between BMI and mBDNF^c^						
Saliva	−0.038	.79	0.096	−0.090 to 0.28	0.092	.30
Plasma	0.33	.020^d^	27.81	−0.24 to 55.87	13.93	.052
Association between plasma and salivary mBDNF^c^						
	0.17	.24	33.95	−10.43 to 78.34	22.02	.13

Abbreviations: BMI, body mass index; CI, confidence interval; K6, Kessler 6; SE, standard error.

^a^Adjusted for age, sex, BMI, and exercise habits in multivariable.

^b^Adjusted for sex, BMI, and exercise habits in multivariable.

^c^Adjusted for age, sex, and exercise habits in multivariable.

^d^Statistical significance was set at *p* < .05.

Table 3 presents the comparison of salivary and plasma mBDNF between men and women. Both univariate (saliva; *p* = .29, plasma; *p* = .53) and multivariate (saliva; *p* = .22, plasma; *p* = .65) analyses revealed no significant differences. Table 4 displays the effect of exercise habits on salivary mBDNF and plasma mBDNF. Neither univariate (saliva; *p* = .51, plasma; *p* = .69) nor multivariate (saliva; *p* = .30, plasma; *p* = .97) analyses indicated any significant differences.

**TABLE 3:** Differences in salivary and plasma mature brain‐derived neurotrophic factor (mBDNF) between men and women.

	Total	Men	Women	*p*‐Value^a^	Adjusted *p*‐value^b^	Effect size^c^ (*r*)
Saliva	1.36 (1.12–1.96)	1.26 (1.12–1.51)	1.44 (1.17–2.18)	.29	.22	0.15
Plasma	1261.11 (242.98)	1272.48 (251.96)	1249.74 (238.31)	.53	.65	0.09

*Note*: Salivary mBDNF was described by median (IQR), and plasma mBDNF was *mean* (*SD*).

Abbreviations: IQR, interquartile range; *SD*, standard deviation.

^a^Analyzed using the Wilcoxon rank‐sum test.

^b^Adjusted for age, BMI, and exercise habits in multivariable.

^c^Effect size of the Wilcoxon rank‐sum test (*r*).

**TABLE 4:** Differences in salivary and plasma mature brain‐derived neurotrophic factor (mBDNF) between participants with and without exercise habits.

	With exercise habits (*n* = 12)	Without exercise habits (*n* = 38)	*p*‐Value^a^	Adjusted *p*‐value^b^	Effect size^c^ (*r*)
Saliva	1.24 (1.11–1.72)	1.37 (1.16–1.96)	.51	.30	0.09
Plasma	1263.72 (251.40)	1260.20 (243.60)	.69	.97	0.06

*Note*: Salivary mBDNF was described by median (IQR), and plasma mBDNF was *mean* (*SD*). We show a scatter plot matrix of the association of mBDNF with each factor (scatter plot and kernel density estimation).

Abbreviations: BMI, body mass index; IQR, interquartile range; K6, the Kessler 6; SD, standard deviation.

^a^Analyzed using the Wilcoxon rank‐sum test.

^b^Adjusted for age, sex, and BMI in multivariable.

^c^Effect size of the Wilcoxon rank‐sum test (*r*).

## 4 DISCUSSION

The novelty of this study lies in the assessment of salivary mBDNF and its relationship with psychological distress in healthy healthcare workers. To enhance the reliability of our findings, we also measured plasma mBDNF and explored its association with psychological distress. Moreover, we investigated the correlations between salivary or plasma mBDNF and factors, such as age, sex, exercise habits, and BMI. Although previous studies have employed ELISA to measure BDNF, most of them focused on total BDNF in blood without distinguishing between pro‐BDNF and mBDNF, and none of them examined mBDNF in saliva. Our study results demonstrated no correlation between salivary mBDNF or plasma mBDNF and psychological distress in healthy healthcare workers. Additionally, no correlation was observed between salivary mBDNF and plasma BDNF, and no associations were found between salivary mBDNF or plasma mBDNF and age, sex, or exercise habits. Additionally, an association between plasma mBDNF and BMI was found only in univariate analysis.

In this study, saliva was successfully used to consistently measure mBDNF. The mBDNF ELISA kit utilized in this study demonstrated high sensitivity, with a lower calibration limit of 0.205 pg/mL. The intra‐assay *CV* and inter‐assay *CV* values for salivary mBDNF were low and stable. This is the first report on the specific measurement of salivary mBDNF in healthy healthcare workers.

Previously, due to the much lower concentrations of salivary BDNF compared to blood BDNF, consistent measurement using commercially available kits has been reported to be challenging as the concentrations often fall below the limit of measurement (Vrijen et al., 2017). Mandel et al. (2011) developed an optimized, highly sensitive, and specific ELISA for salivary BDNF because commercial ELISA kits (Chemicon and Promega) rarely reached minimum detection levels. They reported no correlation between salivary BDNF concentrations (median = 618 pg/mL) and serum BDNF concentrations in 36 healthy volunteers. Subsequently, several studies reported measurements of salivary BDNF using commercially available ELISA kits (R&D, R&D Duoset, Millipore CYT306) (de Souza et al., 2012, 2015; Saruta et al., 2012; Tirassa et al., 2012). Measurements of salivary BDNF levels in healthy young adults (Matsuki et al., 2014; Saruta et al., 2012; Tirassa et al., 2012) showed great variability, with mean values ranging from nine to several hundred pg/mL. In most studies, BDNF levels below the minimum detection threshold were not discarded and were interpreted as true results (de Souza et al., 2012, 2015; Matsuki et al., 2014). Vrijen et al. (2017) examined the association between plasma and salivary BDNF in six healthy volunteers with three commercially available kits (R&D DBD00, LSBio LS‐F2402, Millipore CYT306), but could not measure salivary BDNF. The Millipore kit failed to generate a standard curve using saliva and standards (Vrijen et al., 2017). Similarly, the R&D and LSBio kits had no saliva samples falling within the calibration range, with most samples not exceeding the blank level (Vrijen et al., 2017). Thus, it has been difficult to accurately measure the salivary BDNF levels using commercially available ELISA kits. Notably, the commercial BDNF ELISA kit employed in previous studies used a colorimetric detection method, whereas the commercial mBDNF ELISA kit used in this study employed a luminescent method. The luminescence method is more sensitive than the colorimetric detection method owing to its higher dynamic range and lower background noise. This difference in detection methods may account for the observed high sensitivity and consistent measurement results.

Salivary mBDNF in this study showed a non‐normal distribution. Similarly, Mandel et al. (2011) found variable and non‐normally distributed salivary BDNF concentrations among healthy subjects. Variation is common in salivary protein studies (Mandel et al., 2011), with factors, such as circadian rhythm, flow rate, stress, aging, and infection, known to affect salivary proteins (Rudney, 1995). The influence of the wide range of sample collection times and the non‐normal distribution of age in this study cannot be ruled out.

The present study did not find an association between salivary mBDNF and psychological distress in healthy healthcare workers. BDNF expression in the submandibular gland tissue of humans and mice has been reported to be absent under non‐stress conditions (De Vicente et al., 1998). A study in rats reported that BDNF mRNA is not expressed in the submandibular gland in the absence of stress (Ernfors et al., 1990). However, under stressful conditions, both BDNF mRNA and protein expression in submandibular gland tissue showed an increase (Tsukinoki et al., 2006). Additionally, immobilized stressed rats exhibited a significant rise in BDNF mRNA and protein expression in the submandibular gland, compared to non‐stressed rats (Saruta et al., 2010). In a review by Saruta et al. (2020), they concluded that BDNF mRNA significantly increases after 30 min of stress, whereas BDNF levels decrease after 180 min of immobilization stress compared to non‐stressed rats, indicating enhanced BDNF expression in response to stress.

Our study results also revealed no correlation between plasma mBDNF and psychological distress in healthcare workers. Studies using standardized psychosocial stressors have reported increased serum BDNF levels in healthy participants (Hermann et al., 2021; Linz et al., 2019; Meng et al., 2011). Exercise‐induced acute stress also elevates BDNF in serum and plasma (Huang et al., 2014; Rojas Vega et al., 2006; Szuhany et al., 2015). Our previous study found a negative correlation between plasma BDNF and psychological job stress, suggesting a decrease in plasma BDNF due to job stress (Okuno et al., 2011). Plasma BDNF has been reported to increase in patients with workplace stress‐related adjustment disorders (Buselli et al., 2019). The animal study supports the idea of increased initial BDNF levels as a homeostatic response to stress, with elevated BDNF gene expression in the rat brain following stress exposure (Rage et al., 2002). Both acute and chronic stresses have been shown to raise plasma BDNF levels in rats (Saruta et al., 2010). Serum and plasma BDNF levels are lower in patients with depression than in healthy controls, indicating a negative correlation between the severity of the depression and serum BDNF (Bus et al., 2015; Lee et al., 2007; Molendijk et al., 2011, 2014; Yoshimura et al., 2007). These findings imply that the effect on BDNF depends on the stress levels and the subject status.

Participants exhibited relatively low levels of psychological distress, with a K6 score of 1 (0–3). Therefore, it is reasonable to assume that our study participants were not stressed, and thus, there were no changes in salivary mBDNF. Moreover, this study employed the K6, a shorter questionnaire compared to previous studies (Mitoma et al., 2008; Okuno et al., 2011), instead of the Stress and Arousal Check List used in prior research, which evaluated psychological stress and mental arousal through a set of 13 questions (Mackay et al., 1978). The lack of a correlation between saliva or plasma mBDNF and psychological distress may have been attributed to differences in stress measurement methods, and overall low levels of psychological distress among mentally healthy healthcare workers.

No correlation was observed between plasma and salivary mBDNF in this investigation. Previous studies by Jasim et al. (2018) using capillary isoelectric focus immunoassay in 20 young healthy volunteers reported the detection of salivary BDNF in unstimulated sublingual saliva and stimulated parotid saliva, but no significant correlation was found between saliva and plasma BDNF levels. No association was found between salivary and plasma BDNF in a study using a similar method in 10 young healthy subjects (Jasim, Ghafouri, Carlsson, et al., 2020). Similar to the previous study, no correlation was found between saliva and plasma in the mBDNF evaluated in this study. The reason for the lack of correlation between salivary mBDNF and plasma mBDNF is unclear but may be due to the fact that each is secreted by different cells.

No association between mBDNF and age was found in saliva and plasma. Previous studies have reported that plasma BDNF does not correlate with age (Okuno et al., 2011), whereas others have reported a decrease with age (Lommatzsch et al., 2005). Similarly, in accordance with Mandel et al. (2011), this study found no correlation between age and salivary BDNF concentration, aligning with the previous study.

Moreover, no sex differences in mBDNF levels were observed in either saliva or plasma. In contrast, according to Mandel et al. (2011), women had significantly higher salivary BDNF levels than men. Saruta et al. (2012) reported that salivary BDNF concentrations tended to be higher in women than in men. According to Jasim, Ghafouri, Gerdle et al. (2020), there was no difference in salivary and plasma BDNF concentrations between the sexes. Some studies have revealed that plasma BDNF levels in women are substantially higher than in men (Okuno et al., 2011); however, no significant differences were observed after controlling for body weight (Lommatzsch et al., 2005), showing the existing uncertainty regarding the causes of the variations in results between studies. Platelet BDNF levels have been reported to vary with the menstrual cycle (Lommatzsch et al., 2005), and salivary BDNF in women has been reported to show a pattern similar to that of estradiol with the menstrual cycle. The inconsistent relationship between BDNF and sex may be due to the non‐consideration of the menstrual cycle during sample collection.

No association was observed between salivary mBDNF and BMI, consistent with previous research findings (Mandel et al., 2011). With regards to plasma mBDNF, a negative correlation with BMI was found only in univariate analysis but not in multivariate analysis. Previous studies have reported either no relationship (Okuno et al., 2011) or a negative correlation (Lommatzsch et al., 2005) between plasma BDNF and BMI. Some studies suggest lower serum BDNF levels in obesity compared to normal‐weight individuals (Ceylan et al., 2023), indicating a potential risk for obesity in individuals with lower BDNF levels (Li et al., 2016). However, other studies have found no significant differences in basal BDNF levels between obese and normal‐weight individuals (Sandrini et al., 2018). The association between BMI and BDNF is complex, and findings across studies are inconsistent.

Additionally, we found no differences in salivary and plasma mBDNF levels in participants with or without exercise habits. Wang et al. (2022) performed a meta‐analysis to examine the effect of exercise on BDNF and observed that both acute exercise and long‐term aerobic exercised resulted in increased BDNF levels. Salivary BDNF has been reported to be higher in elite basketball players than in sedentary individuals (Moreira et al., 2018). Within our study sample, exercise status was not assessed, which is a potential limitation in the detailed assessment of the subject's exercise amount, intensity, and duration.

This study had some limitations. The study did not control for the time of sample collection, physical activity, oral medications or supplements, the menstrual cycles of female participants, or genetic polymorphisms of pro‐BDNF and BDNF. Sample quality was not compared to other indicators such as salivary cortisol, amylase, or chromogranin A. The narrow range of K6 scores among participants may have contributed to the lack of correlation between salivary or plasma mBDNF and psychological distress. Future studies with patients with psychiatric disorders and a larger study sample should be performed. Finally, the possibility of other unnoticed confounding factors influencing the results cannot be ruled out.

## 5 CONCLUSION

This study aimed to investigate whether salivary mBDNF could serve as a biomarker of psychological distress in healthcare workers. Salivary mBDNF was reliably measured, but no significant correlation was observed with psychological distress. Additionally, salivary mBDNF levels mirrored those in plasma. It is important to consider that the participants in this study had low K6 scores and a narrow range of values, which may not fully represent the broader population. Consequently, the generalizability of these findings is limited, and this study should be regarded as preliminary. Further studies with larger and more diverse participants, particularly encompassing a wider range of psychological distress, are warranted. Additionally, further studies should explore the potential of salivary mBDNF as a noninvasive biomarker associated with psychiatric disorders.

## AUTHOR CONTRIBUTIONS

All authors made a significant contribution to the work reported, whether that is in the conception, study design, execution, acquisition of data, analysis, and interpretation or in all these areas; took part in drafting, revising, or critically reviewing the article; gave final approval of the version to be published; have agreed on the journal to which the article has been submitted; and agree to be accountable for all aspects of the work.

## CONFLICT OF INTEREST STATEMENT

The authors declare no conflicts of interest.

## FUNDING INFORMATION

This research received no external funding.

### PEER REVIEW

The peer review history for this article is available at https://publons.com/publon/10.1002/brb3.3278.

## CONSENT FOR PUBLICATION

Written informed consent was obtained from all participants involved in the study.

## Data Availability

The data that support the findings of this study are available upon request from the corresponding author. Due to privacy and ethical restrictions, some limitations may apply to the availability of certain confidential data.
